# Outcomes of Intertrochanteric Fracture Fixation Using the Trochanteric Fixation Nail Advanced (TFNA): A Retrospective Analysis

**DOI:** 10.7759/cureus.99507

**Published:** 2025-12-17

**Authors:** Ramprasad Jasti, Prithvi Mohandas, Mahesh K Ragavan, Sunil D Magadam, Umesh Kannadasan

**Affiliations:** 1 Department of Orthopaedics, Madras Institute of Orthopaedics and Traumatology (MIOT) International Hospital, Chennai, IND

**Keywords:** functional outcomes, harris hip score (hhs), intertrochanteric fracture, radiographic union score for hip (rush), trochanteric fixation nail advanced (tfna)

## Abstract

Background: Intertrochanteric hip fractures in the elderly pose a major healthcare burden due to their high morbidity, mortality, and economic impact. The Trochanteric Fixation Nail-Advanced™ (TFNA) Proximal Femoral Nailing System (DePuy Synthes, West Chester, PA, USA), a type of cephalomedullary device, has shown potential for improving outcomes in the management of these fractures. This study aimed to evaluate the functional and clinical outcomes of trochanteric fracture fixation via TFNA.

Methods: A retrospective, single-center observational study was conducted at a tertiary orthopaedic trauma hospital in India and included 368 patients who underwent femoral fixation with the TFNA system between January 2019 and December 2022. Radiological parameters (tip-apex distance [TAD], Radiographic Union Score for Hip [RUSH]), functional outcomes (Harris Hip Score [HHS]), surgical duration, intraoperative blood loss, complication and reoperation rates, and mortality were assessed at six weeks, three months, six months, and one year postoperatively.

Results: The mean age of the patients was 63.8 years, with 60.9% being male. The mean TAD was 14.9 mm at both six months and one year, and the mean RUSH score was 29.8 at six months and 30.0 at one year, reflecting effective fracture healing. The mean HHS was 93.39, with 92.8% achieving excellent functional outcomes. The average surgical time was 50.6 minutes, with mean blood loss of 128.4 mL. The revision rate was low (0.3%), and mortality rate was 1.63%.

Conclusions: TFNA fixation for intertrochanteric fractures yields excellent radiological and functional outcomes, with low complication and revision rates. Optimal implant placement and surgical technique are crucial for successful fracture management.

## Introduction

Hip fractures are a significant healthcare challenge in the elderly population, resulting in substantial pain, increased morbidity and mortality, and financial burden [1]. The worldwide annual incidence of hip fractures is estimated to be 2.6 million by 2025 and is projected to rise to 4.5 million by 2050 due to aging [2]. With an increasing elderly population in developing countries, it is estimated that half of these fractures will occur in Asia. In India, the estimated annual incidence is over 120 fractures per 100,000 persons, which could translate to 0.2 million hip fractures in patients older than 50 years [3,4].

Geriatric intertrochanteric fractures are a major public health concern due to their associated disability, morbidity, mortality, and healthcare costs. The compromised bone quality and unstable nature of these fractures complicate their management [5]. Unstable intertrochanteric hip fractures are estimated to constitute 55% of all hip fractures in the elderly population [6]. These fractures have unique anatomic and mechanical characteristics and are traditionally considered unstable [7]. Stable fractures have an intact posterior dial cortex. Unstable fractures include comminution of the posteromedial cortex, a thin lateral wall, displaced lesser trochanter fracture, subtrochanteric extension, and intertrochanteric fractures [8].

The current literature indicates that intramedullary nailing is one of the most effective surgical fixation options for unstable intertrochanteric fractures, offering superior outcomes compared with arthroplasty [9]. While stable trochanteric fractures can be successfully treated with a sliding hip screw, intramedullary nails are preferred for managing unstable trochanteric and subtrochanteric fractures [10]. Intramedullary nailing, which is particularly effective for unstable patterns such as reverse obliquity fractures, provides the advantage of a minimally invasive approach, reducing blood loss [8].

Comparative studies on extramedullary devices and intramedullary nails for unstable intertrochanteric fractures are scarce. However, available research indicates that intramedullary nails offer superior outcomes. Patients receiving intramedullary fixation have better lower extremity measure (LEM) scores and faster union times. Failures with extramedullary devices often stem from complications in reduction, fixation, and alignment maintenance until fracture healing [11].

Currently, several intramedullary nailing systems are commercially available, including the Trochanteric Fixation Nail-Advanced (TFNA) Proximal Femoral Nailing System (DePuy Synthes, West Chester, PA, USA), the TRIGEN InterTAN Intertrochanteric Antegrade Nail, and the Intramedullary Hip Screw (IMHS) (Smith and Nephew, Memphis, TN, USA). Compared with other designs, the TFNA, which is widely used for trochanteric, subtrochanteric and segmental fractures of the femur, was designed in 2015 to offer increased nail strength and durability [12]. Although clinical data on the TFNA remain limited, recent studies have highlighted low complication rates and high levels of radiological healing associated with its use [13].

This study aims to provide real-world evidence on the functional and clinical outcomes of patients undergoing fixation surgeries for femoral fractures via retrospective data from a tertiary hospital.

## Materials and methods

Study design

A retrospective, single-center, observational study was conducted at a tertiary orthopedic trauma hospital in India. Patients with intertrochanteric fractures who underwent femoral fixation via the TFNA system were evaluated. Patients who underwent femoral fixation via the TFNA from January 2019 to December 2022 were included in this study. Patients who underwent orthopedic surgery other than femoral fixation via the TFNA system and patients who underwent orthopedic surgery before January 2019 were excluded. Data on patient age, sex, comorbidities, intraoperative and postoperative radiological and functional outcomes (including tip-apex distance [TAD], X-ray Radiographic Union Score for Hip (RUSH), and Harris Hip Score [HHS]), operative duration, blood loss, pre- and postoperative hemoglobin (mg/dL), complications, reoperation, and mortality were obtained from each patient's medical records. Clinical and radiological follow-up data at six months and one year after surgery were collected. The study was performed in accordance with the Declaration of Helsinki and was approved by the institutional ethics review board (ECR/1114/Inst/TN/2018/RR-21,MIOTIEC/2023/01, Dated 12th April 2023). Informed consent was exempt or not required because of the retrospective nature of the study.

Surgical Procedure

Patients underwent surgery as early as possible after completing relevant investigations, radiographs, and obtaining anaesthetic clearance between 12 and 36 hours after admission. After the induction of general or regional anaesthesia, patients were positioned on an orthopaedic traction table in the supine position. All patients underwent an intraoperative safety check and antibiotic prophylaxis (ceproxime 1.5 g) prior to making the incision.

The key step in surgery is fracture reduction. The reduction of trochanteric fractures is primarily achieved prior to surgery. Fine adjustments were made intraoperatively. Hence, adequate time needs to be spent on good fracture reduction. A 5 cm longitudinal incision was made proximal to the greater trochanteric with the shaft of the femur. The entry point was taken at the piriformis fossa medial to the greater trochanter, and care was taken not to reach the fracture site but to reach the proximal fragment. This allows a better hold and maintains valgus reduction. The canal was opened with an awl, and serial reaming was performed. The appropriate TFNA nail was inserted into the canal. We prefer 235 mm nails for all unstable trochanter fractures.

Abduction of the leg was performed to obtain valgus at the fracture site such that the proximal calcar was aligned with the distal calcar at the proximal end of the calcar 2 mm medial to the distal calcar, which was called positive reduction. The guidewire is placed to the head such that the tip is pointing toward the foveal capitis, and the wire is placed in the inferior segment in the AP view and posterior segment in the lateral view, which is called foveal centering.

A helical blade was placed through the nail to the femoral head; placement was monitored using fluoroscopy. Fracture site compression was achieved using the system, and the set screw was locked in static mode. Finally, the system was secured with distal screws.

After surgery, all patients received thromboprophylaxis. Blood tests and postoperative X-rays were performed, and patients underwent regular physiotherapy and orthopedic review. Early seating and active mobilization of the operated limb began within the first 24 hours. Controlled gradual weight-bearing was permitted starting the day after surgery.

Study outcomes

The primary endpoints of this study were radiological and functional outcomes measured by the TAD and RUSH scores at six months and one year. This study also evaluated secondary endpoints, including the duration of surgery, blood loss, pre- and post-Hb levels, intra- and postoperative complications, the HHS, mortality and the revision rate at one year.

Statistical analysis

Descriptive statistics are presented as the means (standard deviations, SDs) or medians (interquartile ranges) according to the data distribution. Continuous data are presented as the means and SDs and were compared via Student's t test. Categorical data are presented as numbers and percentages. A p value of less than 0.05 was considered to indicate a statistically significant difference. The statistical analyses were conducted via SPSS software version 20.0 (IBM Corp., Armonk, NY, USA) and Microsoft Excel® 2019 (Microsoft, Redmond, WA, USA)

## Results

In total, 368 patients underwent femoral fixation using TFNA during the study period, and all were included in the study. Among the 368 participants, 224 (60.9%) were males, and 144 (39.1%) were females. The mean age (standard deviation [SD]) at the time of admission was 63.8 (±15.9) years (age range: 20-92 years). The major comorbidities included hypertension (33.1%), followed by diabetes mellitus (28.8%) and coronary artery disease (CAD) (6%) (Table 1).

**Table 1:** Patient demographic characteristics CAD: Coronary Artery Disease; COPD: Chronic Obstructive Pulmonary Disease; SD: standard deviation

Characteristics	(n=368)
Age (Mean±SD)	63.8±15.9
Sex (n, %)	-
Male	224 (60.9)
Female	144 (39.1)
Comorbidities (%)	-
Hypertension	33.1
Diabetes Mellitus	28.8
CAD	06.0
Hyperlipidaemia	02.2
Osteoporosis	01.9
Hypothyroid	01.9
COPD	01.6
Others	15.2

The average duration from admission to surgery ranged from six to 36 hours. Of the two available head element options, a helical blade or a threaded screw, the threaded screw was used in four patients, while the helical blade was used in the remaining cases. The threaded screws were chosen for younger patients with good bone stock. The majority of cases involved fractures in elderly patients aged 60 to 70 years, for whom TFNA helical blades were used. Additionally, in six elderly patients with very severe osteoporosis, TFNA cement augmentation was applied alongside the helical blades

Radiological outcomes

For radiological outcomes, TAD and RUSH scores were measured postoperatively and at six weeks (in 364 and 367 patients), three months (in 362 and 363 patients), six months (in 361 and 362 patients), and one year (in 359 and 360 patients) after surgery. The mean TAD was 15.3 ± 4.0 mm at six weeks, 15.1 ± 4.0 mm at three months, and 14.9 ± 4.0 mm at both six months and one year of follow-up. The mean RUSH score was 29.8 ± 0.78 at the six-month follow-up and 30.05 ± 0.05 at the one-year follow-up (Table 2).

**Table 2:** Radiological Outcomes SD: standard deviation; TAD: tip-apex distance; RUSH: Radiographic Union Score for Hip

Parameters	At six months (mean±SD)	At one year (mean±SD)
TAD Score (mm)	14.9 ± 4.0	14.9 ± 4.0
RUSH Score	29.79 ± 0.78	30.00 ± 0.05

Functional outcomes

The functional results were evaluated via the HHS for the 359 patients at one year post-surgery. The mean (SD) score was 93.39 ± 1.85. The HHS for the 26 patients (7.2) was in the range of 80-89, which was good, and excellent results were reported for 333 patients (92.8%), with a score range of 90-100. No patients with HHS in the poor category (<70) were included (Table 3).

**Table 3:** Functional and clinical outcomes a There was slight variation in sample size for the Harris Hip Score because of missing data for some patients; min: minutes; mL: millilitre; SD: standard deviation

Parameters	(n=368)
Harris Hip Score ^a^	-
Mean(SD)	93.39(01.85)
Good: 80-89 (n, %)	26 (7.2%)
Excellent: 90 -100 (n, %)	333 (92.8%)
Surgical time (min)(mean±SD)	50.61 ± 20.55 (range: 15.00-125.00 mins)
Amount of blood loss (mL) (mean±SD)	128.44 ± 47.35 ml (range: 65-300 ml)
Hemoglobin (g/dl) (mean±SD)	-
Preoperative	11.28 ± 3.10
Postoperative	10.32 ± 1.75
Mortality (n, %)	6 (1.63%)

Surgical duration and amount of blood loss

The average duration of surgery was 50.61 ± 20.55 minutes (range: 15-125 minutes). The average blood loss was 128.44 ± 47.35 ml (range: 65-300 ml). The mean values for preoperative haemoglobin and postoperative haemoglobin were 11.28 ± 3.1 g/dL and 10.32 ± 1.75 g/dL, respectively (p value=0.001) (Table 3).

Revision, mortality and complications (safety)

Only one patient (0.32%) underwent revision surgery due to blade failure, which was addressed with an angled blade plate and cement augmentation. Another patient experienced an increased TAD with sepsis and a subluxed hip. At the one-year postoperative mark, two patients (0.54%) reported a total of three complications, including blade failure, increased TAD, and septic/subluxating hips.

In total, six deaths were reported, all due to non-surgical reasons. Among these, two were attributed to cardiac arrest, two to respiratory causes (one due to bronchopneumonia and one due to atrial flutter), and one was due to old age (Table 3).

## Discussion

The treatment of unstable intertrochanteric fractures, especially in elderly individuals, presents significant challenges. Prolonged immobilization can adversely affect overall outcomes [14]. The literature is divided into the use of intramedullary devices and the use of extramedullary devices for treating unstable hip fractures. A systematic review by Zeelenberg et al. reported statistically significant results favouring intramedullary fixation for endpoints such as the HHS, Parker Mobility Score, Lower Extremity Measure, time to full weight-bearing, superficial infection, nonunion, fixation failure, leg shortening, time to bone healing, and duration of surgery [15]. The average age of the patients in this study was 63.8 years, indicating that most were elderly. The literature indicates that most intertrochanteric fractures in the geriatric population are unstable and have become more complex over the past decade [16].

Our study included radiological and functional outcomes, including TAD, and emphasized the fact that healing without complications such as cut out, varus collapse, and back out. The RUSH was used to assess union in femoral fixation after the use of TFNA.

The TFNA nailing system has advantages in treating unstable trochanteric fractures because of its low failure rates. Failures are caused mainly by cut-out of the head screw or breakage of the head screw/nail screw junction [13]. Lag screw cut-out is a major complication in such surgeries, and the TAD accurately predicts the probability of this complication [12]. A TAD of less than 25 mm significantly decreases the risk of complications, including a reduction in the neck-shaft angle, neck length, and offset, along with better functional scores [17]. Our study reported a mean TAD of 15.7 mm postoperatively and 14.9 mm at six months and one year, which are comparable to the findings of another study using the TFNA, which reported a mean TAD of 20.5 mm and a mean calcar TAD of 24 mm in cases of implant fracture with radiographic nonunion [13]. We found that standard piriformis entry, positive reduction and foveal-centered placement of the blade eventually led to a reduced TAD, thus reducing the number of complications reported earlier. C-arm images of pyriformis entry, positive reduction, and foveal centring.

A significant association was reported between TADs and functional outcomes. A lower TAD score is correlated with better functional scores, improved neck-shaft angles, neck length offsets and fewer implant-related complications of implant failure. The literature suggests that an increase in TAD is related to a decrease in HHS [17]. The HHS is a valid and reliable assessment for evaluating patients' functional outcomes after surgery [18,19]. Among the 368 patients in this study, 26 reported good HHSs (80-89), and 333 reported excellent results (90-99). HHSs below 55 at two and five years, along with a lack of improvement or worsening from baseline within two years, have been identified as predictors of early revision in patients who underwent primary total hip arthroplasty (THA) [20].

Although the literature places considerable emphasis on the importance of the TAD and keeps it 25 mm intraoperatively, very few methods for obtaining a lower TAD have been described. This article is a pilot study that emphasizes practical tips for reducing the TAD, which includes piriformis entry, foveal centring of the blade (inferior and posterior) and positive reduction (valgus reduction) [21].

The RUSH score describes radiographic healing of trochanteric fractures by assessing the presence of bridging calluses at the fracture site. Frank et al. [1] reported that patients with an RUSH score below 18 were 10 times more likely to undergo nonunion reoperation than those with scores higher than 18 (relative risk [RR], 9.9; 95% CI, 4.4-22.7). Similarly, the 18-point threshold was predictive of reoperation for all indications (RR, 2.7; 95% CI, 1.7-4.4) [1,3]. This study revealed RUSH scores of 29.8 and 30.0 at six months and one year, respectively, with only one revision surgery, which signifies that complete union is obtained and that the implants are well maintained throughout the healing phase without any complications. The failures may be attributed to increased TAD distance and blade failure, for which revision with an angle blade plate and augmentation with cement were performed. In the above cases, foveal centering and positive reduction were not performed, as these are cases from the beginning of the series, which may be a possible reason for failure.

The mean surgical duration in this study was 50.61 ± 20.55 minutes (range: 15-125 minutes), comparable to the 59.69 minutes noted in a study of elderly patients using the TFNA in India [14]. A Japanese study reported an average surgical time of 56.8 ± 19.6 minutes (range: 23-123 minutes) [7]. Similarly, a study published in 2022 comparing PFNA and external fixation for intertrochanteric femur fractures reported a mean operative time of 52.7 ± 16.2 minutes (range: 30-95 min) for PFNA procedures [22]. The shortest is its mins. Some patients required mini-open techniques and additional reduction techniques, such as correction of flexion deformities via the use of ball spikes/collinear clamps; in those patients, the surgical time was longer. Shorter operative periods and lower invasiveness correlate with decreased operative blood loss and no significant change in haematocrit postoperatively [14]. The average blood loss in the Japanese study was 89 ± 41 mL (range: 0-245 mL). In patients treated with PFN for unstable intertrochanteric femur fractures, the mean blood loss volume was 173.75 ± 28.64 mL [4]. This study reported a mean blood loss of 128.44 ± 47.35 mL (range: 65-300 mL) in 26 patients, with no blood transfusions needed. Postoperative haemoglobin levels were significantly lower (p=0.001). A study evaluating morbidity and mortality in patients with intertrochanteric fractures treated with the TFNA reported mean preoperative hemoglobin, postoperative hemoglobin, and anemization values of 12.33 g/dL, 10.03 g/dL, and 2.83 g/dL, respectively. More unstable fractures cause more blood loss, with mean losses of 2.95 ± 1.33 g/dL, 3.8 ± 1.5 g/dL, and 2.6 ± 1.05 g/dL in patients with fracture types 31A2, 31A3, and 31A1, respectively [23].

Schneider et al. endorsed the use of TFNA due to the low incidence of postoperative complications, suggesting good outcomes with proper reduction quality and implant positioning [13]. According to Lopez-Hualda, the TFNA group, which had a higher frequency of the most unstable trochanteric hip fractures, reported a higher success rate in achieving full weight-bearing at hospital discharge. Mortality is directly related to the time taken to undergo surgery, so early surgery (within two days of admission) is recommended to improve outcomes and reduce mortality risks [23]. A Korean study also reinforced the use of TFNA for treating intertrochanteric femur fractures [24].

An improvement in outcomes and fewer postoperative complications with TFNA can be achieved with a better learning curve and understanding of the instrument and its details [14].

Among the 368 patients studied, only two experienced complications, while the remaining 366 patients demonstrated excellent functional and radiological outcomes. The reduction loss and complications in these two cases might be attributed to the placement of the blade outside the foveal centring mode. These results emphasize the importance of primary reduction and precise implant placement, particularly achieving minimal TAD through positive valgus reduction and foveal centring, as a practical approach to ensure reproducible and consistent outcomes.

Limitations

This study has a few limitations, including its retrospective design, which may introduce selection bias, and the lack of a control group to compare the outcomes with those of contemporary procedures in a similar population.

## Conclusions

In conclusion, the TFNA nailing system shows promising results in managing unstable intertrochanteric fractures among elderly patients. When combined with effective reduction techniques and accurate implant positioning, TFNA provides significant functional and radiographic benefits, as demonstrated by the positive outcomes observed in the majority of patients in this study. Nevertheless, further prospective studies with control groups are essential to validate these findings and improve the effectiveness of TFNA in various clinical settings.
